# Natural Food Components as Biocompatible Carriers: A Novel Approach to Glioblastoma Drug Delivery

**DOI:** 10.3390/foods13172812

**Published:** 2024-09-04

**Authors:** Arunraj Tharamelveliyil Rajendran, Anoop Narayanan Vadakkepushpakath

**Affiliations:** Department of Pharmaceutics, NGSM Institute of Pharmaceutical Sciences (NGSMIPS), Nitte (Deemed to be University), Mangalore 575018, Karnataka, India; arunraj.21phdp102@student.nitte.edu.in

**Keywords:** food components, polysaccharides, proteins, lipids, glioblastoma, drug delivery, nanocarriers, cancer

## Abstract

Efficient drug delivery methods are crucial in modern pharmacotherapy to enhance treatment efficacy, minimize adverse effects, and improve patient compliance. Particularly in the context of glioblastoma treatment, there has been a recent surge in interest in using natural dietary components as innovative carriers for drug delivery. These food-derived carriers, known for their safety, biocompatibility, and multifunctional properties, offer significant potential in overcoming the limitations of conventional drug delivery systems. This article thoroughly overviews numerous natural dietary components, such as polysaccharides, proteins, and lipids, used as drug carriers. Their mechanisms of action, applications in different drug delivery systems, and specific benefits in targeting glioblastoma are examined. Additionally, the safety, biocompatibility, and regulatory considerations of employing food components in drug formulations are discussed, highlighting their viability and future prospects in the pharmaceutical field.

## 1. Introduction

Glioblastoma, commonly known as glioblastoma multiforme (GBM), is recognized as one of the most aggressive and lethal forms of brain tumors [1]. This malignancy is characterized by its rapid progression and infiltrative nature within the brain, making complete surgical removal challenging [2]. Although there have been significant improvements in multimodal therapy, the outlook for people with GBM remains unfavorable, with a median survival time usually below 15 months [3]. There is an urgent need for better therapeutic methods to combat GBM because of the significant mortality rate that is associated with this terrible disease [4].

The blood–brain barrier (BBB) is a critical protective mechanism that prevents the passage of potentially harmful agents and maintains brain homeostasis by regulating the transit of substances from the bloodstream into the brain. Comprising brain endothelial cells lining the cerebral microvasculature, the BBB acts as a physical and physiological barrier that limits the delivery of drugs to the brain [5]. Additionally, the blood–brain barrier’s selective permeability and its ability to restrict the passage of therapeutic agents into the brain parenchyma significantly hinder the efficacy of conventional drug delivery methods [6]. The BBB poses a significant obstacle to the transportation of chemotherapeutic drugs into the brain, limiting the efficacy of systemic treatments and posing a significant challenge in managing this aggressive form of brain cancer [7]. Physiological barriers such as the BBB and blood–tumor barrier further complicate the management of glioblastoma and underscore the critical need for novel delivery approaches [8]. Overcoming the challenges posed by the BBB is essential for developing innovative drug delivery systems that can improve the outcomes of GBM therapy and enhance patient survival.

The current standard treatment protocol for GBM typically involves a combination of radiation therapy, chemotherapy, and surgical resection [9]. Surgical resection aims to remove as much of the tumor as safely possible, followed by adjuvant therapies to target any remaining cancer cells [10]. Radiation therapy, often administered concurrently with chemotherapy, helps to destroy cancer cells and prevent their regrowth [11]. Chemotherapy, commonly utilizing agents like temozolomide, plays a crucial role in targeting cancer cells and inhibiting their proliferation [12]. Temozolomide, an alkylating agent, is a critical component of chemotherapy regimens for glioblastoma and works by promoting methylation of O6-methylguanine-DNA methyltransferase (MGMT), a DNA repair enzyme associated with treatment resistance [13]. The current treatment choices have limited effectiveness, highlighting the urgent necessity for the development of new potent therapeutic techniques to tackle this lethal disease.

Recently, there have been endeavors to investigate innovative therapeutic strategies for GBM., including the repurposing of renin-angiotensin system modulators and the investigation of herbal medicines as a potential therapeutic intervention [14,15]. These alternative treatment strategies aim to overcome the limitations of conventional therapies and improve patient responses to treatment. Additionally, the development of targeted therapies and immunotherapeutic approaches holds promise in enhancing the efficacy of treatment regimens for glioblastoma [16].

Food components (polysaccharides, proteins, lipids, and phytochemicals) are being explored as potential drug carriers due to their biocompatibility, non-toxic nature, and the potential to cross the blood–brain barrier, offering a promising avenue for therapeutic applications [17,18,19,20]. Employing molecules derived from food presents an intriguing therapeutic option, especially in light of the challenges involved in designing molecules that can effectively penetrate the BBB [21]. Food components, such as peptides derived from casein, have shown promise in crossing models of the intestinal barrier and BBB, highlighting their potential as carriers for drug delivery to the brain [22]. Specific food components, like caffeic acid found in vegetables and fruits, have been shown to penetrate the BBB in humans, indicating the ability of dietary phenolics to traverse this barrier and potentially exert beneficial effects on the brain [23]. The non-toxic and biodegradable nature of food components makes them attractive candidates for drug delivery systems. For instance, carbon dots derived from food sources exhibit promise as drug carriers, although they face challenges similar to those of penetrating the BBB, as many small molecules do [24]. Similarly, hyper-crosslinked polymers (HCPs) have attracted significant attention in the field of material science due to their unique properties, particularly their permanent microporosity achieved through a hyper-crosslinking reaction based on Friedel–Crafts chemistry [25]. This reaction allows for the rapid formation of strong linkages, resulting in a highly crosslinked network that can be advantageous for various applications, including in the biomedical field. One notable advantage of hyper-crosslinked polymers is their ability to incorporate drugs and therapeutic agents into their porous and crosslinked network. This enables controlled release through modulation of porosity, crosslinking degree, and swelling behavior. This feature makes them promising candidates for drug delivery systems, offering a platform for tailored and sustained release of pharmaceutical compounds [26,27]. However, despite their promising attributes, hyper-crosslinked polymers also come with certain disadvantages that must be considered. One notable drawback is the potential lack of biodegradability in some cases, which can pose challenges in terms of long-term compatibility and clearance from the body. Unlike natural carriers that can undergo enzymatic degradation or metabolic processes, some hyper-crosslinked polymers may persist in the biological system, raising concerns about their biocompatibility over extended periods. Additionally, the synthetic nature of hyper-crosslinked polymers may introduce issues related to immunogenicity or toxicity, especially if the materials used in their synthesis are not biologically inert. These factors underscore the importance of thorough biocompatibility assessments and long-term safety evaluations when considering the use of hyper-crosslinked polymers in biomedical applications. Using food-derived materials as biocompatible carriers offers a promising solution for GBM drug delivery. These natural components possess inherent advantages, such as improved biocompatibility and biodegradability, which minimize the risk of adverse reactions and facilitate seamless integration within the body. Moreover, incorporating food-based materials can enhance the solubility and stability of therapeutic agents, overcoming common formulation challenges. By leveraging the inherent properties of food-derived substances, researchers can engineer targeted, controlled-release formulations that enhance the therapeutic efficacy of anti-cancer agents while minimizing adverse side effects. This strategy not only harnesses the power of nature but also aligns with the growing emphasis on green, sustainable solutions in the pharmaceutical industry. As the field of GBM research continues to evolve, exploring food components as biocompatible carriers represents a promising avenue to improve patient outcomes and pave the way for more effective, patient-centric treatment options.

This review delves into recent applications of nanoscopic drug delivery systems that incorporate food components. It explores a variety of nanocarriers, with a particular emphasis on polysaccharides, proteins, and lipids. Food components, including functional small compounds and macromolecules, are integrated into these nanostructures, either as the delivered therapeutic agents or as materials for the carriers. It provides a detailed examination of how these food-derived components are utilized to enhance the efficacy and targeting of drug delivery systems. Polysaccharides, known for their biocompatibility and biodegradability, are highlighted for their role in forming stable and efficient drug carriers. Proteins, with their versatile, functional properties, are discussed for their ability to form nanoparticles that can protect and transport drugs. Lipids, which naturally interact well with biological membranes, are noted for their use in creating lipid-based nanoparticles that improve drug solubility and bioavailability.

Furthermore, this review discusses the clinical potential of these food-derived nanoscopic drug delivery systems, with a specific focus on GBM management. GBM, an aggressive form of brain cancer, presents significant treatment challenges due to the difficulty of delivering therapeutic agents across the BBB. This review examines current research on the ability of these nanocarriers to effectively target and treat GBM, offering insights into their mechanisms of action and therapeutic potential.

In summary, this review highlights the innovative use of food components in developing advanced nanoscopic drug delivery systems. It underscores the importance of these natural materials in improving drug delivery and therapeutic outcomes, particularly in the context of glioblastoma treatment. This review calls for continued research to further understand and enhance these promising strategies.

## 2. Types of Food Components

### 2.1. Polysaccharides

**Figure 1:**
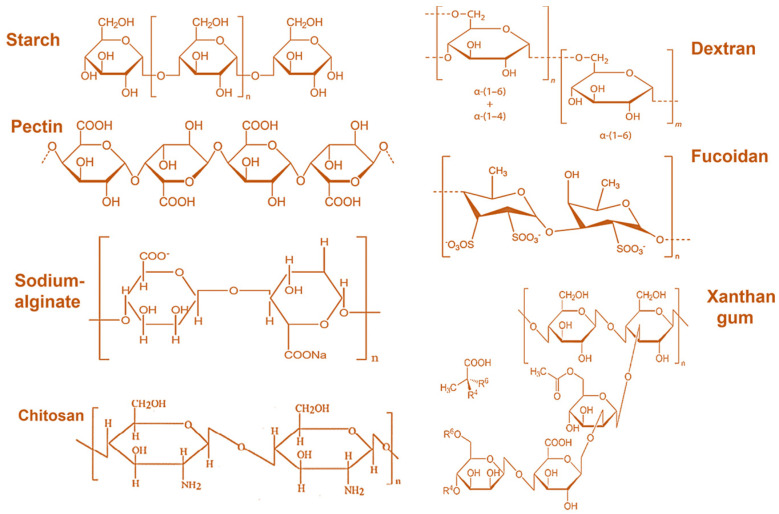
Chemical structure of polysaccharides.

Polysaccharides are intricate carbohydrates with extended chains of monosaccharide units joined by glycosidic bonds. [28]. These natural polymers, such as starch, gelatin, alginate, chitosan, and its derivatives, have attracted substantial attention in drug delivery applications due to their biocompatibility and biodegradability [29]. Polysaccharides offer a versatile platform for developing controlled drug delivery systems, enabling the release of encapsulated drugs at particular sites and times [30]. In order to understand how drugs are released from polysaccharides in drug delivery systems, it is essential to investigate the interactions and behaviors of these biocompatible polymers when coupled with therapeutic agents. One of the primary mechanisms of drug release from polysaccharide-based systems is diffusion. Drugs enclosed within polysaccharide matrices can diffuse out of the polymer network over time, resulting in sustained release profiles [31]. The release rate can be impacted by factors such as the size of the drug molecules, the porosity of the polysaccharide matrix, and the interactions between the polymer and the drug [32]. For instance, marine polysaccharide-based nanoparticles have been developed to release drugs through a controlled diffusion process, ensuring optimal drug concentrations at the target site [33]. Secondly, the swelling mechanism is triggered when the polysaccharide matrix comes into contact with biological fluids. The matrix absorbs the fluids, expands, and creates larger pores or channels through which the drug can be released. Thirdly, degradation involves the enzymatic or chemical breakdown of the polysaccharide matrix. As enzymes or chemical agents in the biological environment degrade the matrix, the structural integrity of the matrix is compromised, leading to the release of the encapsulated drug. Lastly, in polyelectrolyte complexes, ion exchange processes can trigger drug release. These complexes often involve interaction between charged drug molecules and the polysaccharide matrix. When ions in the surrounding biological fluids interact with the matrix, they can replace the bound drug molecules, leading to their release. Each of these mechanisms can be tailored to specific therapeutic needs, providing a versatile platform for drug delivery systems. These polysaccharides can create various drug delivery systems, including hydrogels, nanoparticles, and microparticles, which are pivotal in regulating drug release kinetics [30]. The mucoadhesive properties of marine polysaccharides, such as chitosan, further enhance their effectiveness in drug-delivery systems [34]. Despite their advantages, challenges exist in optimizing polysaccharide-based drug delivery systems. Structural instability of polysaccharides can impact the synthesis process and the performance of drug delivery carriers, necessitating careful design considerations [35]. Strategies like enzyme-responsive, pH-sensitive polymers and microorganism-degradable polysaccharides have been proposed to target drug delivery to specific sites, highlighting the need for innovative approaches to overcome challenges in drug release mechanisms [36]. Figure 1 represents the chemical structure of polysaccharide food components.

#### 2.1.1. Starch

Starch, a crucial component with diverse applications in various industries such as pharmaceuticals, food, textiles, breweries, and distilleries, is sourced from a wide array of plants [37]. Traditional sources like maize, cassava, and potatoes have been extensively studied and utilized for their starch content [38]. However, as countries industrialize, there is an increasing demand for both native and modified starches, which is often met through imports rather than local production. Cereals like maize, wheat, and rice are primary sources of starch for food applications [39]. Starch can be derived from plants’ roots, stems, and seeds, showcasing its versatility in sourcing. Moreover, nonconventional sources of starch, including legumes like jack beans and moth beans, present cost-effective alternatives that warrant further exploration [40]. Furthermore, tubers like Dioscorea remotiflora have been identified as rich starch sources, indicating the potential for alternative starch sources beyond the commonly used ones [41]. Green banana starch, with its unique properties and increasing demand in the food industry, represents another unconventional source gaining attention [42]. Starch-based nanomaterials have attracted considerable interest due to their exceptional biocompatibility and potential application as carriers for drug administration, including nanospheres, micelles, nanogels, and nanofibers. [43]. Starch, a common component found in various food sources, has been widely studied for its applications in drug delivery systems. Waxy starches, such as those found in cereals like rice, corn, and sorghum, have been utilized in different food applications, including as transporters for drug delivery [44]. Starch–lipid or starch–lipid–protein complexes have been identified as potential carriers for accommodating small molecules with low solubility in aqueous media, showcasing the versatility of starch in drug delivery systems [45]. Moreover, resistant starches have been investigated for their health properties and potential to be incorporated into food components, highlighting their role in improving human health and potentially enhancing drug delivery systems [46]. Starch nanoparticles have emerged as a promising tool in drug delivery, with studies focusing on their use in sustained drug release and targeted delivery applications [47]. These nanoparticles have been prepared using different methods, such as precipitation within ethanol as a precipitant, showcasing the diverse approaches to synthesizing starch nanoparticles [48]. Chen et al. developed a new method by combining nanoprecipitation and successive centrifugation to generate starch nanoparticles, which showed less than 50 nm size. The process is simple and eco-friendly and was found to be physically stable [49]. Researchers have investigated the encapsulation of various drugs, such as paclitaxel and curcumin, within starch nanoparticles to enhance their stability and bioavailability [50,51].

Moreover, using starch nanoparticles in controlled drug release systems has been a subject of interest, with modified starch materials serving as carriers and encapsulating agents for preparing nanoparticles [52]. These systems offer controlled release properties, making them valuable for delivering drugs to active sites within the body [53]. Starch nanoparticles have been studied for their potential in various routes of drug delivery, including nasal drug carriers in the form of powders, nanoparticles, or microspheres [54]. The versatility of starch nanoparticles in drug delivery systems is further exemplified by their application in combination therapies, where they have been used to co-deliver multiple drugs for enhanced therapeutic effects due to the functional group availability for binding drug-targeting ligands and sustained release properties [55]. Additionally, the utilization of starch-coated magnetic nanoparticles has demonstrated promise in targeted drug delivery, indicating the potential of starch-based materials in GBM treatment [43]. Starch nanoparticles (SNPs) offer biocompatible, biodegradable drug delivery for glioma treatment, enhancing targeted therapy, crossing the BBB, and enabling multimodal approaches. Ongoing research focuses on optimizing their design and ensuring clinical safety and efficacy. The future of starch-based applications, particularly in drug delivery and treatment, is highly promising due to their biocompatibility, biodegradability, and versatile functional properties. Starch nanoparticles are poised to revolutionize drug delivery systems, offering enhanced stability, bioavailability, and the controlled release of therapeutic agents, including for challenging conditions like glioblastoma. Advances in nanoprecipitation techniques and the development of novel starch-based carriers, such as starch-coated magnetic nanoparticles, are expected to improve targeting precision and efficacy in drug delivery. As research progresses, the focus will likely be on optimizing these systems for clinical applications, ensuring safety and efficacy, and expanding their use in combination therapies and other innovative treatment approaches. The exploration of nontraditional starch sources and their unique properties will also contribute to the development of cost-effective and sustainable solutions in both pharmaceutical and food industries.

#### 2.1.2. Chitosan

Chitosan, a copolymer composed of glucosamine and N-acetyl glucosamine, derived from the alkaline deacetylation of chitin polysaccharide, which is found naturally in algae, fungi, insects, mollusks, and exoskeletons of crustaceans [56]. Chitosan is a cationic polymer that has two groups that could be modified: an amine (NH_2_) and a hydroxyl (OH). As a result, N-linked and O-linked alterations can provide a broad range of chitosan derivatives. Chitosan modification of NPs can improve brain penetration [57]. When it comes to delivering high molecular weight medications, the nose-to-brain pathway shows great promise as an alternative to the conventional BBB crossings. Bettina et al. developed transferrin-conjugated chitosan nanoparticles for protein delivery through the nose-to-brain route. Targeting ligands on nanoparticle surfaces can increase bioavailability by increasing cellular absorption through selective binding and a longer residence period [58]. In the field of theranostics, chitosan nanoparticles have been recognized as a versatile platform for combining diagnostic and therapeutic functions in cancer treatment [59]. Leila et al. used chitosan nanoparticles for encapsulating doxorubicin and superparamagnetic iron oxide nanoparticles (SPIONs), and these nanoparticles were prepared using the ionic gelation method [60]. Lysozyme is the primary enzyme that degrades chitosan in vivo by the hydrolytic action of the acetylated residue. Chitosan degradation rates are inversely related to the degree of crystallinity, which is determined by the degree of deacetylation. Studies have demonstrated that chitosan can be utilized as a food additive, preservative, coating material, and packaging component due to its biocompatibility, biodegradability, and antimicrobial properties [61,62,63]. Chitosan possesses a diverse array of distinctive characteristics that render it a valuable constituent in applications pertaining to food and pharmaceuticals. The future of chitosan-based applications in food and pharmaceuticals is highly promising, driven by its biocompatibility, biodegradability, and antimicrobial properties. In pharmaceuticals, advancements in nanoparticle technology and innovative delivery routes like the nose-to-brain pathway are expected to enhance drug delivery and efficacy, particularly for neurological disorders and cancers. The integration of diagnostic and therapeutic functions in chitosan nanoparticles promises a new era in theranostics. In the food industry, chitosan’s use as a preservative, coating material, and packaging component is anticipated to grow, driven by its natural origin and safety profile, with ongoing research likely to optimize its functional properties and expand its applications.

#### 2.1.3. Alginate

Alginate, a polysaccharide derived from brown seaweed, has garnered significant attention in various industries, particularly in food applications. It serves as a versatile ingredient due to its unique properties such as non-toxicity, biocompatibility, and biodegradability. In the food sector, alginate is extensively utilized as a thickener, emulsifier, stabilizer, and gelling agent [64]. Its wide array of applications includes being a thickening agent in desserts, sauces, and beverages as well as a stabilizer in food products [65]. Moreover, alginate is recognized as being generally safe by regulatory bodies like the USFDA, making it a natural choice for food packing materials [66]. The unique properties of alginate, in combination with other materials like chitosan, have made them attractive as nanocarriers for drug delivery applications [67]. In biomedical and pharmaceutical research, the hydrogelling capacity of alginate has expanded its applications, facilitating the encapsulation of proteins and drugs for controlled release and targeted delivery. [68]. Neshastehriz et al. examined how alginate nanogel co-loaded with gold nanoparticles and cisplatin affected U87-MG human glioma cells in a chemo-radiotherapeutic manner. This combination approach improves therapeutic efficacy and accelerates the rate of apoptosis rather than necrosis [69]. Moreover, alginate-coated nanoparticles have been investigated for their role in improving drug residence time at absorption sites, thereby enhancing drug efficacy and bioavailability [70]. For example, Huang et al. developed magnetic nanoparticles that incorporated a core (doxorubicin-gelatin) and an exterior layer (Fe_3_O_4_-alginate) to function as targeted anticancer drug delivery vehicles. These core–shell nanostructures provide high encapsulation efficiency and controlled release [71]. Alginate interacts with cationic molecules by electrostatic processes and chelates with divalent cations to create hydrogel or nanoparticles, which makes it an appropriate drug carrier [72]. Alginate nanoparticles offer a promising strategy for glioma treatment due to their biocompatibility, ability to encapsulate various therapeutic agents, and potential for targeted delivery, addressing major treatment challenges such as the blood–brain barrier. Despite significant preclinical progress, further research is needed for clinical translation.

#### 2.1.4. Pectin

Pectin is a complex polymer that is present in plant cell walls, especially in fruits and vegetables. It is used in the food and pharmaceutical industries. Numerous studies have been conducted on the structure and characteristics of pectin, demonstrating its use in food items as an emulsifier, thickening and gelling agent, and stabilizer [73]. Extracted from sources like orange and lemon peels, pectin is a valued hydrocolloid due to its health benefits and functional properties, making it a versatile ingredient in the food, cosmetic, medical, and pharmaceutical sectors [74]. The World Health Organization (WHO) and Food and Agriculture Organization (FAO) have categorized polysaccharides like pectin as safe additives for food applications [75]. 

Pectin’s biocompatibility and biodegradability have made it a preferred choice in producing films, hydrogels, and composite materials for applications in food, pharmaceuticals, and biomedical sectors [76,77]. Furthermore, pectin’s role in encapsulating bioactive compounds and probiotics has been acknowledged, showcasing its potential in enhancing the delivery and efficacy of such components in food products [78,79]. 

Research has investigated the use of pectin-based nanoparticles for drug delivery applications, demonstrating their potential in improving therapeutic outcomes. Development of self-assembling pH-responsive nanoparticle platforms based on pectin–doxorubicin conjugates has been conducted for the co-delivery of anticancer drugs, highlighting the versatility of pectin in nanodrug delivery systems [80]. Studies have highlighted the efficacy of pectin in inhibiting cancer cell growth and inducing apoptosis in various cancer cell lines, including brain cancer cells [81]. Modified pectin fractions obtained through pH and temperature treatments have shown enhanced effectiveness against cancer, especially when they are shorter, low-branched, water-soluble, and rich in galactose [82]. Additionally, pectin extracted from guabiroba pulp has demonstrated anticancer effects on human GBM cell lines [83]. Phoebe et al. suggested a targeted medication delivery system that includes a sprayer, bioadhesive hydrogel (pectin), and drug nanocrystals coated in polylactic acid-polyethene glycol that is intended to be injected straight into the brain parenchyma next to the surgical site. Pectin is used for neurological applications, demonstrating biocompatibility both in vitro and in vivo, adhesion to the mammalian brain, and gelling at normal calcium concentrations in the brain [84]. The development of pectin-based nanocarriers represents a significant advancement in drug delivery, paving the way for personalized and effective therapeutic interventions. The future of pectin in therapeutic and biomedical applications is highly promising, with potential advancements in drug delivery systems and personalized medicine. Ongoing research will enhance the functionality of pectin-based nanoparticles, optimizing them for targeted therapy and controlled drug release. Innovations in pectin’s structural modifications and integration into smart drug delivery systems could revolutionize treatments for complex conditions like glioblastoma. As pectin’s biocompatibility and versatility continue to be explored, it is poised to significantly impact the fields of food, pharmaceuticals, and biomedicine.

#### 2.1.5. Dextran

Dextran, a complex branched polysaccharide polymer, is derived from certain microorganisms like bacteria (Leuconostoc spp.). Dextran consists of a main α-D-1,6-glucose-linked glucan with 1,3-linked side chains [85]. The structure of dextran includes α-(1→6) linked glucose residues forming the backbone, with branching occurring through α-(1→2), α-(1→3), or α-(1→4) linkages [86]. This unique structure contributes to the diverse properties and functionalities of dextran, making it suitable for a wide range of applications. Dextran possesses various functional groups that play a crucial role in its interactions and applications. The molecule contains hydroxyl groups, which are abundant in sugar residues and can be substituted with different chemical entities [87]. Additionally, dextran can be modified to introduce specific functional groups for targeted applications. For instance, dextran can be esterified to create dextran ester derivatives with adhesive properties, showcasing the versatility of dextran in functionalization [88]. The presence of functional groups in dextran allows for tailored modifications to suit specific needs in various industries. The applications of dextran are extensive and diverse, ranging from nanotechnology to medicine and beyond. In nanotechnology, dextran has been utilized as a coating material for iron oxide nanoparticles, enhancing their biocompatibility and stability for various biomedical applications [89]. Moreover, dextran-coated semiconductor quantum dots have been developed for bioanalysis and imaging, leveraging dextran’s properties such as hydrophilicity, low nonspecific binding, and biodegradability [90]. These applications highlight the importance of dextran in nanotechnology for creating functional nanocarriers and bioconjugates. The future of dextran holds exciting potential across various fields, driven by its unique structural properties and versatility. As research advances, dextran could revolutionize drug delivery systems through highly targeted and responsive carriers, enhancing treatment precision and efficacy. In personalized medicine, dextran-based nanoparticles could be tailored to individual genetic profiles, improving therapeutic outcomes. Furthermore, dextran’s role in imaging could expand with the development of advanced contrast agents and nanoparticles, leading to better diagnostic tools. In regenerative medicine, dextran’s biocompatibility and hydrogel formation could enable the creation of innovative tissue scaffolds for enhanced tissue repair. Environmental applications also present opportunities, with dextran-based materials potentially improving water purification and reducing plastic waste. Additionally, the development of smart materials and advanced biosensors using dextran could transform diagnostics and responsive technologies. Overall, dextran’s adaptability and functionalization possibilities promise to drive significant advancements in technology and medicine.

#### 2.1.6. Xanthan Gum

Xanthan gum is synthesized by the bacterium Xanthomonas campestris, a versatile biopolymer widely used in various industries, particularly in the food sector [91]. This microbial exopolysaccharide serves as a stabilizer, thickener, emulsifier, and suspending agent in food formulations, contributing to improved texture and stability [92]. Its unique rheological properties, such as high viscosity at low concentrations and pseudo-plasticity, make it a valuable additive in food products [93]. Xanthan gum’s ability to dissolve easily in both cold and hot water, along with its stability in different pH environments, further enhances its utility as a thickener and stabilizer in food processing [94]. In the food industry, xanthan gum is commonly used in gluten-free products, where it acts as a thickening agent, providing the desired texture and mouthfeel [95]. Xanthan gum’s role in drug release systems has also been explored, highlighting its potential in pharmaceutical applications [96]. Moreover, it has been studied for prebiotic potential, indicating its possible use in synbiotic yogurt production [97]. Xanthan gum has been studied for its ability to modify drug release from different delivery systems like tablets, films, hydrogels, and nanoformulations, showcasing its versatility in pharmaceutical applications [98]. Studies have demonstrated that the use of xanthan gum in nanocarriers enables targeted drug delivery and provides a slow and consistent release, thereby enhancing therapeutic effectiveness. Muddineti et al. indicated that xanthan gum-stabilized polyethylene glycol-modified gold nanoparticles have been employed for delivering both hydrophilic and hydrophobic drugs, underscoring its versatility as a drug delivery carrier [99]. Xanthan gum hydrogels have been utilized as drug carriers and controlled release systems, enabling sustained and controlled delivery of various drugs such as caffeine, azithromycin, ibuprofen, and propranolol HCl [96]. Additionally, xanthan gum has been integrated into composite polymeric microsponge-based gel formulations for topical drug delivery, illustrating its potential in improving drug delivery systems [100]. The future of xanthan gum looks bright, with potential advancements across multiple fields. In the food industry, its role in enhancing texture and stability may be refined for new dietary trends. In pharmaceuticals, its use in targeted drug delivery systems and smart hydrogels could lead to more precise and controlled medication. Additionally, its prebiotic potential might drive innovations in functional foods and synbiotic products. Xanthan gum’s versatility and biocompatibility suggest it will continue to be a valuable asset in developing cutting-edge technologies and health solutions.

#### 2.1.7. Fucoidan

Fucoidan, a sulfated polysaccharide found in brown seaweeds, has gained significant attention in recent years due to its potential as a functional food component. Studies have highlighted the diverse range of benefits associated with fucoidan, positioning it as a promising ingredient in various food products [101]. The European Food Safety Authority (EFSA) has classified fucoidan as a novel food, further emphasizing its emergence as a functional food ingredient [102]. Fucoidan has been identified as a bioactive compound with therapeutic effects, including anti-inflammatory, antineoplastic, and immunomodulatory properties [103]. Its potential applications in the food industry span from bakery and dairy products to soups and snacks, showcasing its versatility as a food additive [101]. Its classification as Generally Recognized As Safe (GRAS) by regulatory bodies like the United States Food and Drug Administration (USFDA) further supports its safety for consumption [104]. Studies have also highlighted the potential of fucoidan to act as a prebiotic, promoting the growth of beneficial gut microbiota and improving gut health [105]. The therapeutic effects of fucoidan have been explored in various contexts, including its role in modulating the immune system, promoting gut health, and potentially serving as an adjunct therapy in cancer treatment [106]. Additionally, fucoidan has been investigated for its antioxidant properties and its ability to protect DNA, further underscoring its potential as a functional food ingredient [107]. Fucoidan has shown immense potential as a nanocarrier for various therapeutic applications. Cho et al. developed a fucoidan-based nanocarrier that effectively conjugates with P-selectin-overexpressed neovascular endothelial cells, facilitating the transport of photosensitizers to tumor sites for enhanced photodynamic therapy [108]. This highlights the targeted delivery capabilities of fucoidan-based nanocarriers in cancer treatment. Fucoidan was used as a biocompatible surfactant and surface-coating biopolymer in fucoidan-coated photothermal nanocarriers, proving its versatility in drug delivery systems [109]. Furthermore, fucoidan has been incorporated into nanocarriers for oral delivery systems, showcasing its potential to enhance drug bioavailability and efficacy. Scientists have developed a nanocarrier based on chitosan and fucoidan loaded with berberine, a compound known for promoting intestinal epithelial tight junction integrity, showing promising results for oral drug delivery [110]. This highlights the role of fucoidan in improving the oral bioavailability of therapeutic agents. In the context of photodynamic therapy, scientists have developed a multifunctional nanocomplex comprising fucoidan, polyamidoamine dendrimer, and MnO_2_ for enhanced photosensitizer delivery and oxygen generation, emphasizing the role of fucoidan in improving therapeutic outcomes [111]. Additionally, fucoidan has been explored in combination with chitosan for the delivery of curcumin, showcasing its potential to enhance the therapeutic effects of bioactive compounds [112]. Indrani et al. evaluated the antiproliferative effects of temozolomide and fucoidan on C6 glioma cells. The synergistic combination promotes apoptosis induction and changes in the downregulation of inflammatory genes [113]. The future perspective for fucoidan as a functional food ingredient and therapeutic agent is promising, driven by ongoing research and technological advancements. As a novel food component with established safety profiles and a broad range of bioactivities, fucoidan’s potential extends beyond traditional applications. Future research is likely to delve deeper into optimizing fucoidan’s therapeutic efficacy through novel delivery systems, such as advanced nanocarriers and targeted drug delivery platforms. Innovations in nanotechnology could enhance fucoidan’s role in personalized medicine, especially in cancer treatment, by improving the precision and effectiveness of therapies. Additionally, exploring fucoidan’s interactions with the gut microbiome and its prebiotic effects could unlock new avenues for promoting overall health and preventing disease. The development of functional food products incorporating fucoidan could cater to a growing demand for health-enhancing dietary components. As regulatory frameworks evolve and more clinical evidence emerges, fucoidan’s integration into mainstream health and food industries is expected to increase, paving the way for its broader application and acceptance.

### 2.2. Proteins

Protein-based nanoparticles have been extensively utilized as carriers for delivering various chemicals and biomolecular drugs, including anticancer drugs and therapeutic proteins, showcasing their versatility and effectiveness in drug delivery [114]. Various proteins, such as ferritin/apoferritin protein cages, plant-derived viral capsids, small heat shock protein (sHsp) cages, albumin, soy and whey proteins, collagen, and gelatin, have been characterized and utilized for drug delivery systems, highlighting the diverse applications of proteins in this field [115]. Caged protein nanoparticles offer desirable features for drug delivery, such as optimal sizes for endocytosis, non-toxic biodegradability, and the ability to functionalize at different interfaces using protein engineering tools [116]. Protein-based drug delivery systems have been recognized for their specific mechanisms of action, high potency, and potential to deliver therapeutic proteins effectively. Natural proteins, such as chaperonin-GroEL, have shown promise as smart hydrophobic drug delivery vehicles for tumor therapy, leveraging the various functionalities of proteins for effective drug delivery [117]. 

#### 2.2.1. Whey Protein

Whey proteins and caseins are the two primary types of milk proteins. Whey protein isolate (WPI) is a significant by-product of whey proteins that is derived from cheeses with a protein content above 90%. Its excellent gelling and emulsification capabilities have made it a popular material for encapsulating carrier materials [118,119]. Furthermore, whey protein isolate possesses exceptional nutritional value and exhibits remarkable bioavailability due to its elevated levels of key amino acids. The primary components of WPI are β-lactoglobulin and α-lactalbumin, which possess remarkable protective film-forming abilities [120]. Maryanne et al. designed a whey protein nanoparticle with a photoactive component for the treatment of glioma, and it is designed by a spray drying technique. Whey protein nanocarriers hold promise for targeted drug delivery to tumor cells, improving bioavailability and lowering treatment resistance [19]. To enhance the stability and bioavailability of marine carotenoid-fucoxanthin, Wang et al. created a nanocomplex employing whey protein isolate as the core and Ca^2+^ crosslinked flaxseed gum as the polysaccharide shell. Moreover, the nano complex was effective against glial PC12 cells and increased cytotoxicity with concentration. By interfering with lipid metabolism, the encapsulated drug increased reactive oxygen species (ROS), damaged mitochondria and controlled programmed cell death [121]. The future of WPI in drug delivery is promising, especially for targeted cancer therapies. Advances in nanoparticle technology and encapsulation methods are set to improve WPI-based carriers’ efficacy and specificity. Researchers are exploring new combinations and optimizations to enhance stability, release profiles, and targeting precision. WPI could play a crucial role in developing more effective, individualized cancer treatments as these innovations evolve.

#### 2.2.2. Casein

Dietary proteins, such as casein, have emerged as promising carriers for drug delivery applications due to their abundance of active sites for functionalization, enhanced biocompatibility, easy availability, and pH-dependent swelling behavior, enabling controlled release of cytotoxic agents in response to the acidic microenvironment of cancer cells [122]. This pH-responsive behavior is particularly advantageous for targeted drug delivery to specific tissues or cells. A fascinating characteristic of casein is that it can self-assemble into micellar structures in an aquatic environment. It is also amphiphilic. Colloidal calcium phosphate functions as a bridge to keep different subunits together through their phosphate groups. These micellar structures are composed of all four subunits bound together by hydrophobic attractive forces [123]. Bindhya et al. prepared a multifunctional protein–inorganic hybrid nanocarrier that is based on superparamagnetic calcium ferrite nanoparticles and is hybridized with milk protein casein. The nanocarrier is further functionalized with glutamic acid and co-loaded with the natural anticancer drug thymoquinone for glioma. The hybrid nanosystem showed 87.4% encapsulation efficiency. Drug release at the cancer site is due to the response to acidic pH and external magnetic field, and it exhibited superior anticancer activity on U87 cells compared to the free thymoquinone [124]. Nanoparticles have been modified with enhancers to enhance their capacity to permeate through brain barriers. Gao et al. formulated menthol-modified casein nanoparticles loaded with 10-hydroxy camptothecin for glioma targeting [125]. Utilizing these nanosystems provides a novel and promising method for addressing brain tumors. The innovative use of casein’s self-assembling micellar structures and pH-responsive behavior opens avenues for highly specific and controlled drug release mechanisms, particularly in the acidic microenvironment of tumors. Continued research into optimizing these protein-based nanocarriers, including enhancing their stability, loading capacity, and targeting precision, could revolutionize treatment protocols for various cancers, including gliomas. Furthermore, integrating novel functionalization, such as magnetic properties or brain-barrier-permeating agents, could significantly improve the efficacy and safety of these systems. As we advance, interdisciplinary approaches combining materials science, biotechnology, and pharmacology will be crucial in developing next-generation drug delivery platforms that address current limitations and offer personalized therapeutic solutions for complex conditions like brain tumors.

#### 2.2.3. Soy Protein

**Figure 2:**
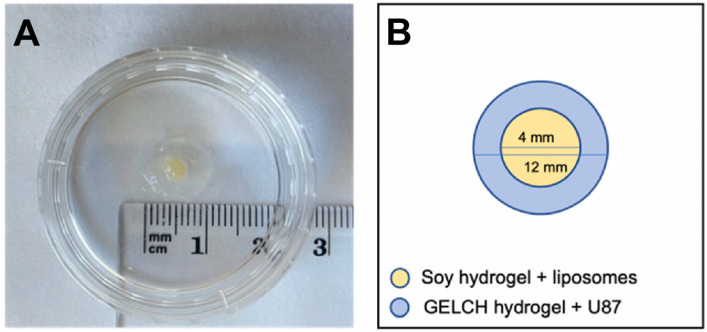
(**A**) Image and (**B**) graphical demonstration of a 3D model hydrogel composed of gelatin-chitosan (GELCH) and U87-MG cells, at the center of which an 18 % soy protein isolate hydrogel containing doxorubicin-loaded liposome was added [135].

Soy protein is a valuable component in both animal and human diets due to its nutritional benefits and functional properties. It is considered a complete protein as it contains all essential amino acids and other macronutrients essential for health [126]. Research indicates that soy proteins can contribute to lowering blood cholesterol levels and reducing the risks of breast cancer and atherosclerosis, thereby promoting human health [127]. Moreover, soy protein has a well-balanced amino acid composition and is particularly rich in lysine, making it a valuable source of protein [128]. Soy protein nanoparticles have shown promise in drug delivery systems due to their unique properties and potential applications. These nanoparticles, derived from soy protein, offer advantages such as solubility in aqueous environments, making them suitable for various drug delivery scenarios [129]. Soy protein nanoparticles have been synthesized for nutraceutical and drug encapsulation purposes, leveraging the abundance and wide utilization of soy protein as a plant-based protein source [130]. The formation of nanoparticle aggregates from soy protein isolate has been achieved through specific treatments, highlighting the feasibility of utilizing soy protein in nanoparticle production [131]. Controlled partial enzymatic hydrolysis has been shown to be an efficacious strategy for devising and constructing multifunctional soy protein nanoparticles [132]. Furthermore, soy protein nanoparticles have been explored for target-specific drug delivery by conjugating them with compounds like folic acid, enhancing their potential for precise drug delivery applications [130]. These nanoparticles can be tailored to control particle size, surface area, and surface properties to optimize drug delivery efficiency and achieve site-specific actions [133]. The unique amino acid composition of soy protein facilitates interactions with hydrophobic drugs, enabling the effective packaging of drugs within soy protein nanoparticles [134]. Viale et al. designed an implantable in situ-gelling gel in the absence of crosslinkers for post-recurrence of GBM. The gel system based on soy protein isolates hydrogels with doxorubicin-loaded liposomes, and it is capable of release in a sustained manner to avoid intracranial hypertension or other adverse reactions [135]. Figure 2 demonstrates the 3D model hydrogel containing a cocktail of food components such as soy protein isolate, chitosan, gelatin and lipids, providing more biocompatibility and the structure of hydrogel that fits the post-surgical cavity properly [135]. Further exploration and optimization of soy protein nanoparticles in GBM treatment could lead to significant advancements in addressing this challenging disease.

#### 2.2.4. Gelatin

Gelatin, a versatile protein derived from collagen, finds extensive applications in the food industry due to its functional properties. Gelatin serves various roles such as a gelling agent, stabilizer, film-forming material, and emulsifier in food products [136]. Traditionally sourced from pigs and cattle, gelatin has seen innovations with alternative sources like chicken skin and fish, offering comparable functionalities [137,138]. The process of obtaining gelatin involves the hydrocolloid extraction of collagen fragments, which transform into gelatin through a structural change from a triple helix to a single helix [139]. Gelatin can be sourced not only from cows but also from other animals like sheepskin, fish scales, and fish byproducts [140,141]. Gelatin’s unique properties, such as its ability to improve food quality and act as a stabilizer in ice cream, make it a valuable component in various food products [142]. Gelatin’s functional properties, including gelling, film-forming, emulsifying, and foaming, make it a versatile ingredient in food products [143]. Gelatin’s role as a water-soluble polymer that enhances elasticity, consistency, and stability in foods further solidifies its importance in food formulations [144]. Gelatin nanoparticles have shown promise as a targeted drug delivery system, particularly in the treatment of GBM. Studies have demonstrated the potential of gelatin nanoparticles functionalized with tumor-targeting peptides in delivering CRISPR/Cas9 to glioblastoma cells [145]. Additionally, research has highlighted the effectiveness of gelatin nanoparticles loaded with cardamom extract as a targeted drug delivery system for treating glioblastoma. The prepared nanoparticles were evaluated as a drug delivery system for treating GBM and were discovered to eliminate human U87MG GBM cells with high efficacy [146]. Furthermore, the development of gelatin nanoparticles for the postsurgical treatment of glioblastomas, such as curcumin-loaded zeolite Y nanoparticles embedded in electro-spun polycaprolactone and gelatin nanofibers, showcases advancements in utilizing nanotechnology for targeted therapies [147,148]. Gelatin nanoparticles have also been explored in combination treatments. The development of matrix metalloproteinase (MMP)-sensitive size-shrinkable gelatin-gold fabricated nanoparticles offers a novel approach for tumor microenvironment-responsive penetration and the diagnosis of glioma. These nanoparticles are engineered to react to MMPs, enzymes that are highly expressed in the tumor microenvironment, resulting in a reduction in size to facilitate enhanced penetration and drug delivery. The system entails the creation of small-sized gold nanoparticles on MMP-2 degradable gelatin nanoparticles, enabling pH-triggered cargo release and active targeting for glioma-specific diagnosis and therapy [149]. This innovative approach underscores the potential of nanotechnology in revolutionizing cancer theranostics by leveraging the unique features of the tumor microenvironment to optimize treatment outcomes. A novel nano-in-nanofiber delivery system for Temozolomide has been developed to achieve sustained release and enhanced cellular uptake by U87MG cells, particularly in the context of glioma treatment. This system involves embedding polymeric nanoparticles within electrospun nanofibers, enabling controlled drug release and improved drug delivery efficiency. By encapsulating Temozolomide within this composite delivery system, the technology aims to enhance the therapeutic efficacy of the drug through sustained release profiles and increased cellular uptake, specifically targeting U87MG cells associated with glioma [150]. Future research on gelatin nanoparticles for glioblastoma could aim to enhance targeting precision and drug release control by integrating advanced functionalization and stimuli-responsive features. Combining gelatin with other materials might improve treatment efficacy and reduce side effects. Validation through in vivo studies will be crucial for advancing these therapies into clinical applications.

#### 2.2.5. Zein

Zein, a prolamine alcohol-soluble protein derived from corn, has garnered significant attention for its versatile applications in the food and pharma industry. Zein has been utilized to produce biodegradable polymeric films that act as coatings to safeguard food products from spoilage [151]. These films have been shown to be effective in delaying rancidity in nuts and preserving the quality of fresh-cut apple slices [152,153]. Moreover, zein has been employed as a carrier for encapsulating various hydrophobic food components such as lipids, vitamins, flavorings, and antioxidants, making it a valuable material for food packaging applications [154]. Additionally, zein has been investigated for its potential in drug and nutrient delivery systems, with zein-based micro- and nanoparticles being explored for these purposes [155]. Zein nanoparticles have shown promise in targeted drug delivery for GBM treatment. Research has demonstrated the successful delivery of curcumin to GBM cells using curcumin-loaded zein nanoparticles that crossed the BBB [156]. Additionally, scientists have developed zein-polydopamine nanoparticles functionalized with the G23 peptide showing the ability to cross an in vitro BBB model and penetrate GBM tumor spheroids [157]. The use of zein nanoparticles in drug delivery systems offers advantages such as enhanced stability and bioavailability of drugs, improved tissue penetration, and targeted delivery to tumor cells. This approach has the potential to enhance treatment efficacy against GBM and other cancers. Nanoparticle-based drug delivery systems, including those with zein nanoparticles, represent a cutting-edge strategy that can improve therapeutic outcomes and reduce side effects [158]. Future research could focus on optimizing the synthesis and functionalization of zein nanoparticles to enhance their targeting precision and drug delivery efficiency. Innovations in this area may lead to more effective treatments with reduced side effects and improved patient outcomes. Additionally, integrating zein nanoparticles with emerging technologies, such as personalized medicine and advanced imaging techniques, could further refine their application in GBM therapy. Overall, the continued exploration and development of zein-based systems could significantly impact the future of cancer treatment, particularly for challenging brain tumors.

#### 2.2.6. Albumin

Albumin, a protein commonly found in various food sources, plays a significant role as a nutritional component. Studies have highlighted the nutritional value and functional properties of albumin in different food proteins such as ovalbumin, bovine serum albumin, and β-lactoglobulin [159]. Research has shown that albumin is the most abundant and nutritionally important component among various proteins, indicating its potential as a valuable food component [160]. Furthermore, albumin, along with other proteins like whey protein isolate, has been utilized as an emulsifier in food systems, emphasizing its role in enhancing the stability and characteristics of food emulsions [161]. In the food industry, albumin has been recognized for its antioxidant activity and high nutritional value, making it a desirable component in food formulations [162]. Additionally, albumin and other protein fractions like glutelin have been identified as favorable nutrition and functional additives in the food industry, showcasing their potential for various food applications [163]. The utilization of albumin extends to the development of plant-based products, edible packaging films, and bioactive compound carriers, highlighting its versatility and potential to meet consumer demands for high-quality plant protein resources [164]. Moreover, albumin has been explored for its ability to form interpolymeric complexes, demonstrating its versatility in creating delivery systems for bioactive ingredients in food products [165]. Albumin nanoparticles have shown promise in targeted drug delivery for glioblastoma therapy. These nanoparticles can penetrate the blood–brain barrier (BBB) and target glioma cells through specific mechanisms, such as SPARC- and gp60-mediated biomimetic transport [166]. Research has demonstrated the efficacy of tumor-targeted multifunctional albumin-based nanoparticles in treating U87 human GBM cells both in vitro and in vivo [167]. Additionally, albumin nanoparticles, exemplified by paclitaxel-loaded Abraxane^®^, have been approved for cancer therapy, highlighting their potential in targeted drug delivery [168]. Studies have indicated that albumin nanoparticles can accumulate in tumor tissues through passive and active targeting mechanisms, underscoring their therapeutic potential in cancer treatment [169]. The unique properties of albumin as a carrier in nano drug delivery systems have been utilized for tumor-targeted delivery to glioblastoma cells, enhancing drug efficacy [170]. Shariatnasery et al. explored combining albumin nanoparticles with other agents for enhanced therapeutic outcomes in GBM. For example, the synergistic effect of microRNA and albumin-bound nanoparticles has shown promise in inhibiting GBM cell proliferation [171]. Moreover, the design of dual-peptide-functionalized albumin-based nanoparticles has demonstrated efficient tumor-targeted drug delivery in GBM cells [172]. In conclusion, albumin nanoparticles offer a cutting-edge approach in GBM therapy, providing targeted drug delivery, BBB penetration, and enhanced therapeutic efficacy. Their multifunctional nature and ability to specifically target glioma cells hold significant potential for improving treatment outcomes in this challenging form of brain cancer.

### 2.3. Lipids

Lipids are essential components of food, serving as structural and functional elements in biological systems. They are present in a variety of food sources, including animal products like meat, dairy, eggs, and fish, as well as plant sources such as soybeans. These dietary lipids are crucial for providing energy, essential fatty acids, and lipid-soluble nutrients necessary for human health [173,174,175]. The interactions between lipids and other food components significantly influence food quality and functionality. Table 1 illustrates the class of lipids, their characteristic nature, examples and their applications.

**Figure 3:**
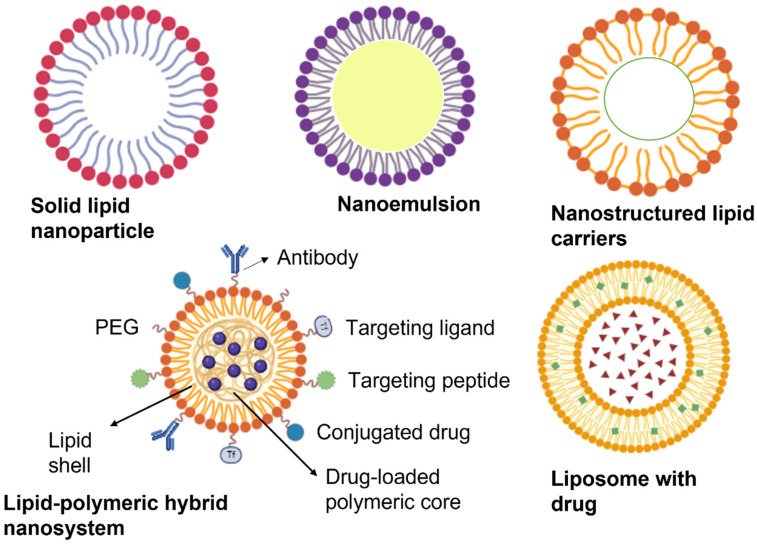
A graphical representation of various types of lipid nanocarrier systems [183,184,185].

Lipids can interact with proteins, starches, and other major food components during processing, affecting the overall structure, stability, and bioavailability of lipophilic food components. Modifying the microstructure and physicochemical properties of foods can influence the bioavailability of dietary lipophilic components [179,180]. Moreover, lipid-based nanoemulsions have been investigated as effective delivery systems for encapsulating lipophilic bioactives in food products. Nanoemulsions provide a transparent and stable matrix for incorporating lipid-soluble bioactives, thereby enhancing their solubility, stability, and bioavailability in aqueous-based food and beverages. This technology offers a promising approach for food fortification and improving the nutritional quality of food products [181]. Lipid nanocarriers enhance the delivery of therapeutic agents across the BBB and target tumor cells more effectively [182]. Several types of lipid nanocarriers, such as liposomes, solid lipid nanoparticles (SLNs), and nanostructured lipid carriers (NLCs), have been developed. Figure 3 demonstrates the different types of lipid-based nanocarrier systems.

Liposomes are spherical vesicles with a phospholipid bilayer capable of encapsulating both hydrophilic and hydrophobic drugs, while SLNs are composed of solid lipids, offering controlled drug release and improved stability. NLCs, a blend of solid and liquid lipids, provide better drug loading capacity and release profiles compared to SLNs. The advantages of lipid nanocarriers include enhanced permeability and retention (EPR) effect, which allows for greater accumulation in tumor tissues, and the ability to protect encapsulated drugs from degradation [186,187,188]. Lipid-based vesicles can encapsulate therapeutic agents, protecting them from degradation and enhancing their delivery to the brain tumor site [189]. Rustad et al. developed pH-responsive liposomes that have shown potential in utilizing the acidic tumor microenvironment of GBM for targeted drug delivery. These liposomes can release their payload in response to the acidic conditions present in the tumor, thereby enhancing the efficacy of chemotherapy agents against GBM cells [190]. Additionally, strategies involving the co-delivery of small-molecule inhibitors and RNA therapeutics using integrin receptor-selective liposomes have demonstrated improved therapeutic benefits by targeting multiple pathways involved in GBM progression [191]. Advancements in nanotechnology have facilitated the engineering of liposomes for the delivery of various therapeutic molecules, including siRNA, chemotherapeutic agents, and photodynamic therapy agents, across the BBB to reach GBM cells [192]. The delivery of mRNA holds significant promise for therapeutic interventions. Numerous studies have delved into the utilization of different delivery systems to enhance the efficacy of mRNA in targeting glioblastoma cells. Kim et al. constructed a nanocomplex utilizing cationic liposomes decorated with a monoclonal antibody to deliver anti-MALAT1 siRNA for RNA interference therapy of glioblastoma [193]. Additionally, the incorporation of ionizable lipid nanoparticles has shown promise in facilitating the delivery of circular RNA for enhanced lung cancer immunotherapy, indicating the versatility of lipid-based systems in mRNA delivery for cancer treatment [194]. Moreover, the use of lipopolymer-lipid hybrid nanoparticles has been shown to efficiently deliver mRNA-based therapeutics for the treatment of various diseases, further underscoring the potential of lipid-based carriers in mRNA delivery [195]. This approach highlights the versatility of liposomes in targeted gene delivery for combating glioblastoma. These innovative approaches have led to enhanced drug accumulation in GBM tumors and improved therapeutic outcomes in preclinical models. Table 2 provides a brief introduction to the different classes of lipid-based nanocarriers for drug delivery, mentioning their structural properties and general applications with examples.

Various natural lipid materials are used in the formulation of lipid-based drug delivery systems for GBM treatment due to their biocompatibility, biodegradability, and ability to form various delivery structures like liposomes, emulsions, and nanoparticles. Here are some natural lipids commonly used in drug delivery.

#### 2.3.1. Lecithin

Lecithin, a natural emulsifier derived from soybeans, is widely used in the food industry for various purposes. It serves as an emulsifier, lubricant, viscosity reducer, anti-spattering agent, and wetting and release agent in food products such as salad dressings, desserts, margarine, chocolate, and baked goods [216]. Additionally, lecithin stabilizes fat, improves texture, and enhances the quality of food products [217]. Lecithin has also been shown to decrease hyperlipidemia and influence lipid metabolism when used as a dietary supplement [218]. Furthermore, lecithin’s antioxidant properties make it beneficial for improving the oxidative stability of food products, such as surimi gel [219]. Utilization of soy lecithin in drug delivery systems has shown promising outcomes for improving the efficacy of treatments for glioblastoma. Soy lecithin-derived liposomal delivery systems offer advantages such as efficient encapsulation, controlled release, and targeted delivery of therapeutic agents to specific sites, including brain tumors like GBM. Incorporating soy lecithin in lipid-polymer hybrid nanoparticles further enhances the drug delivery platform, showcasing the potential for advancing targeted drug delivery strategies for combating GBM [220,221]. The future of soy lecithin in both food technology and drug delivery is promising. In food, it could further enhance product quality and shelf life while improving lipid metabolism. In drug delivery, soy lecithin-based systems, including liposomes and hybrid nanoparticles, show great potential for advancing targeted treatments for glioblastoma and other conditions, with ongoing research aiming to refine their effectiveness and targeting precision.

#### 2.3.2. Phosphatidylcholine 

Phosphatidylcholine (PC), a major component of cell membranes, is widely used in liposome formulations due to its biocompatibility and ability to mimic natural cell membranes. Liposomes incorporating PC have been shown to enhance drug delivery efficiency and improve therapeutic outcomes in cancer therapy, including targeting GBM [222]. The use of PC-based liposomes offers advantages such as improved stability, controlled release, and enhanced biocompatibility, making them suitable carriers for delivering therapeutic agents to target sites, including brain tumors like GBM [223]. The future of phosphatidylcholine (PC)-based liposomes in glioblastoma multiforme (GBM) treatment looks promising with potential advancements in targeted delivery, combination therapies, and overcoming the blood–brain barrier. Tailoring these liposomes for personalized medicine and ensuring their long-term safety and efficacy will be crucial. Continued innovation and research could lead to more effective, individualized treatments and broader accessibility for GBM patients.

In conclusion, liposomal drug delivery systems offer significant potential for enhancing the treatment of GBM by improving the targeted delivery of therapeutic agents to the brain tumor site. By leveraging the unique properties of liposomes, such as their capacity to encapsulate a wide range of drugs and their potential for surface functionalization, researchers are paving the way for more effective and targeted therapies against this devastating disease. As discussed above, a summary of various food components and their drug delivery applications are listed in Table 3.

## 3. Biocompatibility and Immunocompatibility of Major Food Components Used in Drug Delivery

The biocompatibility and immunocompatibility of polysaccharides, proteins, and lipids are crucial considerations for their use as food components in glioma treatment. These natural biomaterials are inherently biocompatible, making them ideal candidates for drug delivery systems that minimize adverse reactions and promote safe therapeutic outcomes. Research indicates that chitosan exhibits immunostimulatory activity by triggering the release of a wide range of cytokines, chemokines, and growth factors from innate immune cells [233]. Despite its immunostimulatory properties, low molecular weight chitosan has been found to possess excellent biocompatibility and anti-inflammatory effects, making it suitable for tissue adhesive applications. Moreover, chitosan’s high molecular weight contributes to its biodegradability and biocompatibility, which are crucial factors in wound healing processes [234].

Studies have demonstrated the biocompatibility and non-immunogenicity of starch-based polymers, showcasing their suitability for a wide range of biomedical applications. Starch-based materials have been proposed for use in biomedical applications such as partially degradable bone cement and controlled release systems, highlighting their potential in advancing healthcare technologies [235]. Alginate, a biodegradable copolymer of guluronic acid and mannuronic acid, has gained significant attention in biomedical applications due to its remarkable biopharmaceutical properties, including pH sensitivity, biocompatibility, biodegradability, mucus adhesiveness, non-toxicity, and non-immunogenicity [236]. Pectin is commonly utilized in various industrial applications, similar to alginate, due to its biocompatibility, biodegradability, and lack of immunogenicity [237]. Pectin has been categorized as a carbohydrate-based bioink, alongside materials like alginate, agarose, cellulose, and chitosan, within the realm of 3D bioprinting technologies for regenerative medicine [238]. The biocompatibility and non-immunogenicity of dextran have positioned it as a preferred material for nanoparticle-based drug delivery systems and imaging modalities. Dextran-coated iron oxide nanoparticles have been identified as highly biocompatible and functional for conjugation with targeting molecules, enabling precise delivery in cancer therapeutics and imaging applications [239]. Additionally, dextran has been utilized in smart responsive hydrogel systems for bone regeneration and controlled drug release, showcasing its potential in regenerative medicine and tissue engineering [240]. Moreover, xanthan gum has been investigated for its role in enhancing the mechanical properties of hydrogels, demonstrating its ability to bind with other polymer chains through hydrogen bonds and electrostatic interactions [241]. This interaction mechanism contributes to improving the mechanical toughness and fatigue resistance of hydrogels, showcasing xanthan gum’s potential in developing durable materials for various applications. The compatibility of xanthan gum with other polymers further highlights its versatility in enhancing material properties. Studies have shown that fucoidan exhibits low toxicity and immunogenicity, making it a promising candidate for cancer therapy due to its ability to induce apoptosis in cancer cells and modulate immune responses. The biocompatibility of fucoidan, coupled with its immunomodulatory effects, positions it as a valuable natural compound for potential applications in cancer treatment and immunotherapy [242].

Proteins also demonstrate high biocompatibility. Whey protein demonstrates antioxidant and anti-inflammatory characteristics as well as bioactivities, including immunomodulatory properties [243]. The intrinsic properties of casein that make it suitable for the food industry, expanding its properties without compromising its biocompatibility and lack of toxicity [244]. Albumin, a naturally occurring plasma protein, is non-immunogenic and can bind various drugs, enhancing their solubility and stability [170]. Gelatin, derived from collagen, is biodegradable and supports cell adhesion and proliferation, making it an excellent material for drug delivery [245]. Zein is a food-grade protein that is generally thought to be less immunogenic than animal proteins like collagen. It was discovered that, even at high concentrations, zein did not cause the generation of TH1 and TH2 cytokines or the proliferation of certain T cells. Zein, known for its biocompatibility, can form stable nanoparticles for controlled drug release [246]. These proteins’ natural roles in the body contribute to their low immunogenicity and compatibility with human tissues.

Lipids, including those used in liposomes and solid lipid nanoparticles, are integral to cell membranes and thus exhibit excellent biocompatibility [247]. Lipid-based carriers can encapsulate both hydrophilic and hydrophobic drugs, protecting them from degradation and facilitating their transport across biological barriers, including the BBB. The biocompatibility of lipids ensures minimal toxicity and adverse immune responses, which is critical for the treatment of sensitive tissues such as the brain [248]. The immune compatibility of these materials requires careful evaluation. Comprehensive preclinical and clinical studies are necessary to assess the immunogenicity of these natural biomaterials. Polysaccharides, proteins, and lipids must be thoroughly tested for any potential to elicit immune reactions, particularly in long-term applications.

In conclusion, polysaccharides, proteins, and lipids offer promising biocompatibility and immunocompatibility for use as food components in glioma treatment. Their natural origin and functional properties support their safe application in drug delivery systems. Ongoing research and rigorous testing are essential to fully understand and mitigate any immunogenic risks, paving the way for their effective and safe use in clinical settings.

## 4. Harnessing the Future of Food-Based Drug Delivery for Glioblastoma

The promising potential of food-based carriers has opened exciting new avenues for improving GBM treatment outcomes. Derived or semisynthetic polymers from food components can indeed enhance drug delivery and improve the permeability of the blood–brain barrier (BBB). These modifications can optimize the inherent properties of the basic food components for better therapeutic efficacy. Firstly, attaching ligands or targeting moieties that can bind to receptors on the BBB facilitates transport across the barrier. Secondly, optimizing the particle size and surface charge to favor transcytosis or passive diffusion. Finally, nanocarriers can be designed to carry multiple drugs or therapeutic agents simultaneously, allowing for combination therapies that can be more effective against GBM. 

The commercial repurposing of food-based materials, particularly nanocarriers derived from polysaccharides, proteins, and lipids sourced from food waste, offers a multifaceted approach to cost reduction, waste management, and bioremediation [249]. Utilizing these natural and biodegradable substances as nanocarriers not only provides a sustainable and cost-effective alternative to synthetic carriers but also helps in the valorization of food waste, turning a potential environmental burden into valuable resources [250]. This approach aligns with the principles of a circular economy, reducing landfill waste and lowering the carbon footprint associated with food disposal. Additionally, the functional properties of these biopolymer-based nanocarriers can be leveraged in various industries, including pharmaceuticals, cosmetics, and food preservation, thereby opening new market opportunities and driving innovation in waste management technologies. By integrating such practices, industries can achieve enhanced sustainability, improved resource efficiency, and contribute to broader environmental remediation efforts [251]. For instance, gelatin, a natural protein derived from collagen found in animal bones, skin, and connective tissues, is a prime example of repurposing food waste into valuable nanocarriers. This protein is widely available from the byproducts of the meat and leather industries, making it an excellent candidate for sustainable practices. Gelatin is prized for its biocompatibility, biodegradability, and non-toxic nature, which makes it ideal for pharmaceutical and biomedical applications [252]. Another example is Chitosan derived from the shells of crustaceans. Chitosan is used in drug delivery systems due to its biocompatibility and biodegradability. It can be obtained from shrimp or crab shell waste, turning what would be a disposal issue into a valuable resource for pharmaceuticals and food preservation [56]. Pectin is extracted from fruit peels, particularly citrus and apple peels, and can be used to create nanocarriers for the delivery of nutrients or drugs. It is a natural gelling agent that also has applications in the food industry [253]. Whey Protein is a byproduct of cheese production, whey protein can be transformed into nanoparticles for use in nutraceuticals and pharmaceuticals. These protein nanoparticles are effective in encapsulating and delivering bioactive compounds [254]. Lecithin is extracted from soybean or egg yolk waste; lecithin is a phospholipid used in the formulation of liposomes and other lipid-based nanocarriers. These are widely employed in the delivery of drugs and nutrients due to their ability to enhance bioavailability and protect sensitive compounds [255]. Fatty acids are derived from various food waste sources such as spent coffee grounds or fish processing waste; fatty acids can be used to form solid lipid nanoparticles or nanostructured lipid carriers for controlled drug release and targeted delivery [256]. 

In conclusion, valuable products from food waste, industries not only create high-value products but also contribute to sustainable waste management and environmental conservation. This approach aligns with the principles of a circular economy, where waste materials are continuously reused and recycled, reducing the overall environmental impact. Ongoing research is exploring innovative ways to further enhance the biocompatibility, stability, and targeted delivery capabilities of these natural materials. By leveraging the inherent properties of common food components, scientists are working to develop even more effective drug delivery systems that can selectively target glioblastoma tumors while minimizing adverse effects. As this field continues to evolve, we can look forward to the emergence of transformative therapies that harness the power of food to combat this devastating form of brain cancer, ultimately improving prognosis and quality of life for patients.

## 5. Conclusions

In conclusion, food components represent a promising class of biocompatible carriers for drug delivery to GBM and other types of cancer. Their natural origin, biodegradability, safety profile, and potential for functionalization make them attractive candidates for enhancing drug delivery to the tumor site while minimizing systemic toxicity. Preclinical studies have demonstrated the efficacy and safety of food-component-based drug delivery systems for GBM treatment, and ongoing clinical trials hold promise for improving therapeutic outcomes in patients. However, several challenges must be addressed to facilitate their translation into clinical practice. Continued research efforts are warranted to optimize food-component-based drug delivery systems and realize their full potential in GBM therapy.

## Figures and Tables

**Table 1:** Class of lipids and their applications.

Class of Lipid	Characteristics	Examples	Applications	References
Phospholipids	Amphiphilic, forms bilayers, good biocompatibility	Lecithin, Phosphatidylcholine	Liposomes, micelles, nanoemulsion	[176,177,178]
Glycolipids	Contains carbohydrate moiety, can interact with cell membranes	Gangliosides, Cerebrosides	Targeted drug delivery, vaccine delivery	
Fatty acids	Simple structure, hydrophobic, used to enhance lipophilicity of drugs	Stearic Acid, Oleic Acid	Prodrugs, lipid nanoparticles, emulsion	
Sterols	Provides stability to lipid bilayers, enhances membrane permeability	Cholesterol	Liposomes, solid lipid nanoparticle	
Glycerides	Major storage form of fats, good solubility properties, biocompatible	Triglycerides, Diglycerides	Lipid emulsions, solid lipid nanoparticles, nanoemulsions	
Sphingolipids	Structural lipids, involved in cell signaling, membrane structure	Sphingomyelin, Ceramides	Liposomes, solid lipid nanoparticles, targeted delivery	
Cationic lipids	Positively charged, interacts with negatively charged nucleic acids for delivery	DOTAP (1,2-Dioleoyl-3-trimethylammonium propane), DOTMA (N-[1-(2,3-dioleyloxy) propyl]-N,N,N-trimethylammonium chloride)	Gene delivery systems, lipoplexes, lipid nanoparticles	
Neutral lipids	Uncharged at physiological pH, useful in forming stable complexes	Dioleoylphosphatidylethanolamine (DOPE), Distearoylphosphatidylcholine (DSPC)	Uncharged at physiological pH, useful in forming stable complexes	
Anionic lipids	Negatively charged, often used to increase the surface charge and stability	DMPG (Dimyristoylphosphatidylglycerol), DPPG (Dipalmitoylphosphatid-ylglycerol)	Liposomes, lipid nanoparticles, nanoemulsions	
PEGylated lipids	Lipids conjugated with polyethylene glycol, improves circulation time	DSPE-PEG	Stealth liposomes, lipid nanoparticles, micelles	

**Table 2:** Classification of lipid-based nanocarriers for drug delivery systems and their application with examples.

I Class of Emulsion Systems			
Class	Subtypes/Examples	Applications	References
Conventional Emulsions	O/W Emulsions (creams), W/O Emulsions (Butter, ointments)	Food, cosmetics, pharmaceuticals	[196]
Multiple Emulsions	W/O/W Emulsions (Cosmetic formulations), O/W/O Emulsions (Specialized drug delivery)	Controlled release, protection of active ingredients	[197]
Microemulsions	O/W Microemulsions (Transdermal delivery systems), W/O Microemulsions (Cosmetic formulations)	Enhanced solubilization, penetration of active ingredients	[198]
Nanoemulsions	O/W Nanoemulsions (Nutraceuticals), W/O Nanoemulsions (Cosmetics)	High stability, enhanced bioavailability, For both hydrophilic and hydrophobic drug delivery	[199]
Self-Emulsifying Drug Delivery Systems (SEDDS)	SEOFs (Oral drug delivery), SMEDDS (Oral drug formulations)	Enhanced oral bioavailability of poorly water-soluble drugs	[200]
**II Class of Vesicular System**			
Liposomes	Small Unilamellar Vesicles (SUVs), Large Unilamellar Vesicles (LUVs), Multilamellar Vesicles (MLVs), Stealth Liposomes, Immunoliposomes, Cationic Liposomes	Drug delivery, gene therapy, targeted delivery	[201]
Niosomes	Span 60, Tween 20	Drug delivery, cosmetic formulations	[202]
Transferosomes	Phospholipids with edge activators	Transdermal drug delivery	[203]
Ethosomes	High ethanol content vesicles	Transdermal and dermal delivery of drugs	[204]
Cubosomes	Nanostructured liquid crystalline particles	Drug delivery, cosmetics	[205]
Cochleates	Solid particulate made up of continuous large lipid bilayer sheets rolled up in a spiral structure with no internal aqueous phase	Hydrophobic and amphipathic drug delivery	[206]
Phytosomes	Complexes of phospholipids and phytochemicals	Improved bioavailability of plant extracts	[207]
Sphingosomes	Sphingolipids-based liposomes	Targeted drug delivery, cancer therapy	[208]
Iscoms	Immune stimulating complexes composed of antigen, phospholipid and cholesterol	Vaccine delivery	[209]
**III Lipid Particulate System**			
Solid Lipid Nanoparticles (SLNs)	Glyceryl behenate, Compritol 888 ATO	Drug delivery, controlled release, targeting specific tissues	[210,211]
Nanostructured Lipid Carriers (NLCs)	Oleic acid, Stearic acid	Improved drug loading, reduced drug expulsion, increased stability	[212]
Liposomes	Phosphatidylcholine, Cholesterol	Encapsulation of hydrophilic and lipophilic drugs, targeted delivery, gene therapy	[213]
Lipid–Drug Conjugates (LDCs)	Fatty acid conjugates, Lipid–peptide conjugates	Enhanced drug stability, improved bioavailability, targeted drug delivery	[214]
Lipospheres	Composed of a solid hydrophobic fat core stabilized by a monolayer of phospholipid	Oral, parenteral delivery, gene therapy	[215]

**Table 3:** Classification of food components, loaded ligands or drugs, biological evaluation methods, and study findings.

Class of Components	Food Components/Derivatives	Drug/Ligand	In Vitro/In Vivo Model	Main Findings	Reference
Polysaccharides	Starch	Iron oxide nanoparticle	RAW264.7 cells were used for the macrophage uptake study. 9L glioma cells were used for tumor induction. Male Fisher 344 rats were used for plasma pharmacokinetic analyses.	Superparamagnetic iron oxide nanoparticles were coated with starch material. Exhibits good biocompatibility. It enhanced magnetic tumor targeting.	[224]
	Chitosan	Silibinin	C6 glioma cells	Developed a sustained-release drug delivery system. C6 glioma cells internalized the drug-loaded chitosan nanoparticles. Silibinin showed toxicity toward C6 cells. Apoptotic genes like Bax and caspase3 expression was increased.	[225]
	Alginate	U87-MG glioblastoma cells	Cisplatin and gold nanoparticles	Synthesized the SPIO@AuNP-Cisplatin-Alginate (SACA) nano complex, composed of an SPIO core, a gold shell, and an alginate coating. Gold nanoparticles and cisplatin were used as potential radiosensitizers. Combination therapy induces more apoptosis than necrosis.	[226]
	Pectin	Etoposide, Olaparib	Human astrocytes cells, U87 GBM cells, male CD-1 NuNu mice (10–11 weeks)	Developed pectin-based bioadhesive hydrogel and drug nanocrystals coated with PLA-PEG for post-surgical localized delivery to brain tumors. Etoposide and Olaparib with high drug loading demonstrated in vitro stability and drug release over 120 h. It showed in vitro and in vivo biocompatibility.	[84]
	Dextran	Paclitaxel	Murine glioma cells (C6) Wistar rat	RVG29 peptide was used as a ligand. Micelles prepared from the dextran provide the possibility of crossing BBB. Tumor growth is suppressed.	[227]
	Xanthan gum	Curcumin	Rat model	Xanthan gum was used as a mucoadhesive polymer for intranasal-to-brain delivery. It showed good stability and controlled release. Xanthan gum-coated liposome exhibited higher drug distribution in the brain	[228]
	Fucoidan	Temozolomide	C6 Cells	Fucoidan enhances the cytotoxicity of temozolomide against C6 cells. Molecular docking and simulation studies proved that temozolomide and fucoidan had the potency to bind to the inflammatory proteins through hydrogen bonds and noncovalent interactions. Dual combination promoted apoptosis.	[113]
Protein	Whey Protein	Aluminium phthalocyanine chloride	GBM grade IV cellline: U87MG cells	Low-cost protein drug delivery system. The hydrophobic drug was successfully encapsulated. Excellent morphological and physicochemical properties. Apoptosis was observed. Desirable for photodynamic therapy.	[19]
	Casein	10-Hydroxycamptothecin	Brain capillary endothelial cells (BCEC), Glioblastoma cells (C6), BALB/c nude mice (male, 4–5 weeks, 18–22 g)	Menthol was used as a brain-targeting ligand. Simple preparation method. Good drug loading capacity. Produced higher cytotoxicity with C6 cells. Promoted late apoptosis. Low systemic toxicity.	[125]
	Soy Protein	Doxorubicin	U87-MG cells	Fabricated implantable in situ gelling gel for the treatment of recurrent GBM. It is biodegradable and showed drug release in a sustained manner.	[135]
	Gelatin	Cardamom extract	U87-MG cells	Cardamom-loaded gelatin nanoparticles were prepared by using a two-step desolvation method. The polymer–extract ratio decided the size of the nanosystem. Showed good stability. A total of 70% of the drug is encapsulated.	[146]
	Zein	Temozolomide RVG29 peptide	U87 -MG cells	Temozolomide-loaded zein nanoparticles fabricated with a ligand RVG29. Showed biocompatibility and brain targeting abilities.	[229]
	Albumin	Temozolomide Hyaluronic acid (HA)	U87 MG cells	HA-conjugated albumin nanoparticles loaded with temozolomide were prepared. Optimized nanoparticles by using box bhenken response surface design. Enhanced temozolomide concentration in the brain due to the targeting ability of HA.	[230]
Lipids	Lecithin	Docetaxel Lactoferrin	U87 MG cells and Swiss albino mice	Lactoferrin was used as a ligand. Solid lipid nanoparticles were prepared using emulsification and solvent evaporation methods. Increased targeting potential in the brain.	[231]
	Phosphatidylcholine	Temozolomide and Vincristine. Lactoferrin and RGD.	U87 MG and T98G cells BALB/c nude mice	Temozolomide and vincristine-coloaded nanostructured lipid carriers were formulated for GBM combination therapy. Exhibited sustained-release behavior, high cellular uptake, high cytotoxicity and synergy effects, increased drug accumulation in the tumor tissue, and obvious tumor inhibition efficiency with low systemic toxicity.	[232]

## Data Availability

No new data were created or analyzed in this study. Data sharing is not applicable to this article.
